# MXene/Doxorubicin Complex-Loaded Supramolecular Hydrogels for Near Infrared-Triggered Synergistic Cancer Therapy

**DOI:** 10.34133/bmr.0163

**Published:** 2025-04-09

**Authors:** Seung Min Yang, Hanseo Bae, Seong-Jong Kim, Mungu Kim, Sang Hoon Hong, Hyunsik Choi, Sei Kwang Hahn

**Affiliations:** Department of Materials Science and Engineering, Pohang University of Science and Technology (POSTECH), Pohang, Gyeongbuk 37673, Republic of Korea.

## Abstract

Photothermal therapy (PTT) has attracted great interest due to the high spatial precision and reduced general toxicity compared to conventional cancer therapies. However, PTT often faces challenges such as incomplete tumor eradication and collateral damage to healthy tissues. Here, we report an injectable MXene–doxorubicin (MD) complex-loaded supramolecular hydrogel (MDGel) for dual synergistic cancer therapy of near-infrared (NIR) PTT and chemotherapy. MDGel is prepared by the host–guest interaction between gelatin–cyclodextrin (GE-CD) and hyaluronic acid–adamantane (HA-AD), facilitating the efficient dispersion of MD complexes in the hydrogel. NIR irradiation triggers the PTT and the release of doxorubicin with increasing temperature. In vitro therapeutic effect is confirmed by achieving nearly 80% cancer cell death via the synergistic effect, compared to the single-modality treatment. In vivo tumor inhibition (68.9% volume reduction) is further validated in skin cancer-bearing model mice with no substantial negative side effect. With its prolonged retention, NIR light-controlled release, and localized therapeutic effect, the MDGel system would provide a notable paradigm as a versatile platform for dual synergistic cancer therapy.

## Introduction

Cancer therapy remains a leading cause of morbidity and mortality worldwide, driving the continuous development of innovative therapeutic platforms that aim to enhance efficacy while minimizing systemic toxicity [1]. Conventional treatments, such as surgery, chemotherapy, cryotherapy, and immunotherapy, often face significant limitations, including inefficient tumor targeting, off-target effects, and systemic side effects [2,3], which compromise patient outcomes. The limitations have spurred the development of other therapeutic approaches, such as photothermal therapy (PTT), photodynamic therapy (PDT), and sonodynamic therapy, to improve the therapeutic effect while reducing adverse effects. Among them, PTT has emerged as an alternative or complementary approach to the conventional cancer treatments [4]. PTT uses photothermal agents to convert near-infrared (NIR) light into localized heat, productively inducing tumor cell death with minimal invasiveness [5]. PTT has demonstrated considerable potential in preclinical studies, offering high spatial precision and reduced general toxicity compared to systemic therapies [6,7]. Nevertheless, PTT alone is often insufficient to achieve complete tumor eradication, as the prolonged treatment duration can cause collateral damage to healthy tissues with the survival of residual tumor cells due to the uneven heat distribution or inadequate thermal penetration [8–10]. Accordingly, these challenges highlight the need for synergistic systems that combine PTT with other therapeutic modalities to maximize antitumor efficacy [11].

MXene (Ti₃C₂T*ₓ*) (MX), a 2-dimensional conductive transition metal carbide [12,13], has emerged as a promising candidate for PTT due to its strong NIR absorbance [14], high photothermal conversion efficiency, and versatile surface chemistry [15,16]. These properties make MX highly effective in generating localized heat under NIR irradiation [17–19], enabling the efficient tumor ablation [20]. Additionally, MX’s large surface area and tunable chemistry allow for facile functionalization and loading of therapeutic agents [21] as a platform for dual-mode cancer therapy [22,23]. For examples, MX was used for the photothermal and electrical stimulation co-therapy [24], PTT and enzymatic dynamic therapy accompanied by photoacoustic imaging [25], and synergistic sonodynamic/chemo dynamic therapy [26]. However, the direct use of MX in cancer therapy has been limited by challenges such as the aggregation in the physiological environment, the reduced stability, and the difficulty in combining its photothermal properties with long term-controlled drug delivery [27].

**Fig. 1.**
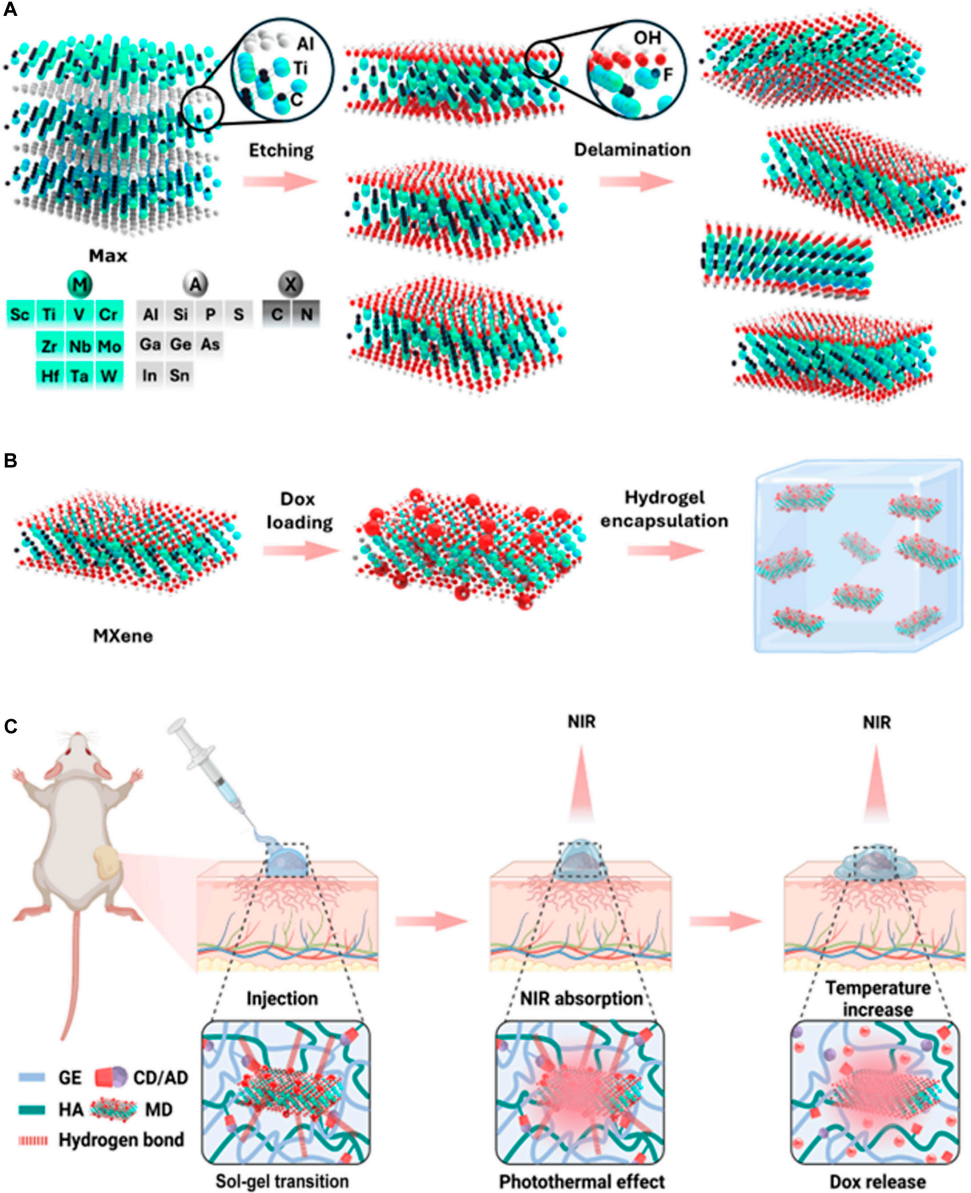
Schematic illustration of the MDGel system. (A) Preparation of the delaminated MX nanosheets and the possible elements for M, A, and X in MAX phase. (B) Encapsulation of MD complexes into the supramolecular hydrogel of GE-CD and HA-AD. (C) Injectable supramolecular hydrogel containing MD complexes and the application to skin cancer therapy via the synergistic photothermal and chemotherapy (created with BioRender.com).

Here, we report a promising injectable MXene–doxorubicin (MD) complex-encapsulated supramolecular hydrogel (MDGel) system for synergistic cancer therapy (Fig. 1). The supramolecular hydrogel matrix is prepared by the dynamic host–guest interaction of gelatin–cyclodextrin (GE-CD) and hyaluronic acid–adamantane (HA-AD) [28,29]. This supramolecular hydrogel can be injected through a syringe and adapted to the tumor shape because of its sol–gel transition upon shear forces. The incorporation of GE into the hydrogel matrix also promotes tissue regeneration and wound healing at the tumor site, addressing the critical need for post-treatment recovery [30,31]. Within the hydrogel matrix, NIR irradiation consistently elevated the temperature close to 56 °C, denoting robust PTT performance and resulting in approximately 80% controlled drug release. After that, we assess the in vitro synergistic effect of the MDGel system in comparison to single-modality treatments. In vivo monitoring over 7 d reveals the 68.9% reduction in tumor size in the group of MDGel with NIR, highlighting the precise tumor targeting and effective suppression of tumor growth. Finally, histological analysis is also carried out to validate the significant reduction in the cell density and increased apoptosis without significant inflammatory responses on the skin surface for NIR light-triggered dual-mode cancer therapy.

## Materials and Methods

### Materials

Sodium hyaluronate with a molecular weight (MW) of 100 kDa was obtained from Lifecore Co. (Chaska, MN). GE from porcine skin, hyaluronidase from *Streptomyces hyalurolyticus*, phosphate-buffered saline (PBS; pH 7.4), sodium periodate, ethyl carbazate, sodium cyanoborohydride and 4-(4,6-dimethoxy-1,3,5-triazin-2-yl)-4-methyl morpholinium chloride (DMTMM), anhydrous dimethyl sulfoxide (DMSO), 4-dimethylaminopyridine (4-DMAP), 1-adamantane acetic acid (Ad-AA), di-*t*-butyl dicarbonate (BOC₂O), MAX precursor (Ti_3_AlC_2_), hydrochloric acid (37 wt %), and lithium fluoride (LiF) were purchased from Sigma-Aldrich (St. Louis, MO). 3A-Amino-3A-dexoy-(2AS,3AS)-b-cyclodextrin hydrate (β-CD-amine) was obtained from Tokyo Chemical Industry (Tokyo, Japan), and 2-(*n*-morpholino)-ethanesulfonic acid (MES) monohydrate was obtained from Georgiachem (Norcross, GA). Doxorubicin hydrochloride (Dox) was obtained from Cayman Chemical (Ann Arbor, MI). Dulbecco’s modified Eagle’s medium (DMEM), fetal bovine serum (FBS), and antibiotic–antimycotic solution were purchased from Invitrogen (Carlsbad, CA). Cell counting kit-8 (CCK-8) was purchased from DoGenBio (Korea), and the murine melanoma cell line (B16F10) was purchased from the Korean Cell Line Bank (Seoul, Korea). TUNEL (terminal deoxynucleotidyl transferase-mediated deoxyuridine triphosphate nick end labeling) assay kits were obtained from Abcam (London, UK).

### Synthesis and characterization of supramolecular GE-CD/HA-AD hydrogel

GE was modified with β-CD-amine via amide linkages using DMTMM as a coupling reagent in a MES buffer (pH 5.5, 100 mM). GE (1 g) was dissolved in the MES buffer at a concentration of 20 mg/ml, followed by the addition of β-CD-amine (3.84 g). Once fully dissolved, DMTMM (468 mg) was added to the reaction solution and the mixture was stirred at room temperature for 24 h. To purify the product, dialysis was performed sequentially against 100 mM sodium chloride solution for 2 d and distilled water for 1 d. The resulting product was lyophilized to obtain GE-CD in the powder form. HA-AD was synthesized via esterification following the previously established protocol. Briefly, HA-tetrabutylammonium salt (HA-TBA; 150 mg, 0.21 mmol repeating units, 1 equiv), 4-DMAP (18.9 mg, 0.15 mmol, 0.7 equiv), and Ad-AA (123 mg, 0.63 mmol, 3 equiv) were dissolved in 7.5 ml of DMSO under argon atmosphere. After the addition of BOC₂O (22.9 mg, 0.11 mmol, 0.5 equiv), the reaction mixture was stirred at room temperature for 24 h. The crude product was purified via dialysis against 20% DMSO solution for 2 d, 0.1 M sodium chloride solution for 2 d, and distilled water for 1 d. The purified product was lyophilized for 3 d to obtain HA-AD as a white powder. The injectable supramolecular hydrogel was synthesized by the host–guest interaction between GE-CD and HA-AD. To assess the properties of self-assembled hydrogel, GE-CD (200 mg/ml) and HA-AD (50 mg/ml) solutions were dissolved in PBS and mixed at a 1:2 ratio for 15 s according to the previous report [28]. The successful conjugation of GE-CD and HA-AD was confirmed by ^1^H nuclear magnetic resonance (NMR; DRX500, Bruker, Germany), and the chemical modifications were validated by Fourier transform infrared (FT-IR) spectroscopy (Agilent Technologies, Santa Clara, CA).

### Synthesis of MX nanosheets

Ti₃C₂T*ₓ* MX nanosheet was prepared by the minimally intensive layer delamination method by optimizing the reproducibility and material purity. Initially, 29.6 ml of concentrated hydrochloric acid (37% w/w) was diluted with deionized (DI) water to prepare 60 ml of a 9 M HCl solution, which was added with 5 g of LiF. The mixture was sonicated to ensure the complete water dissolution of LiF, forming a homogeneous etching solution. Subsequently, 3 g of Ti₃AlC₂ MAX phase powder was gradually introduced to the etching solution over 15 min. The reaction mixture was stirred vigorously at 30 °C for 24 h to facilitate the selective etching of the Al layer and delamination of MAX phase into MX sheets. The resulting slurry was collected by centrifugation and repeatedly washed with DI water to remove the residual salts and reaction by-products. Washing and centrifugation were continued until the pH of the supernatant reached about 6 and the solution exhibited a characteristic dark green color, reflecting the successful formation of Ti₃C₂T*_x_*. The resulting MX clay was dispersed in DI water and then sonicated and centrifuged to collect single-layer MX powder, which was stored at −20 °C for further use.

### Preparation and characterization of MX, Dox, and MD

The crosslinking of injectable supramolecular hydrogel with MD complexes was optimized to prevent aggregation by excess Dox loading or rapid addition. MX in DI water at the predetermined concentrations of 0.4 and 0.6 mg/ml was chosen for their PTT performance and biocompatibility. Independently, Dox was dissolved in DI water to prepare a stock solution. The Dox solution was added dropwise to the MX solution with continuous stirring to promote uniform interaction. The reaction mixture was allowed to stabilize, ensuring the formation of MD complexes via the electrostatic interaction. Unbound Dox was removed by centrifugation at 7,000 rpm for 10 min, and the supernatant was discarded. The pellet containing MD complexes was resuspended in DI water to maintain consistency. To determine the optimal loading condition, Dox concentrations were systematically varied from high to low relative to the MX concentration. The successful drug loading was assessed by energy-dispersive x-ray spectroscopy (EDS) analysis. The target zeta potential was approximately 0 mV, indicating balanced electrostatic interaction within the complexes.

The size and zeta potential of MX, Dox, and MD complexes were measured by dynamic light scattering (DLS) and electrophoretic light scattering (ELS) (ZSU3200. Malvern Instruments, UK). Measurements were conducted at 25 °C in DI water, and each condition was tested in quintuplicate (*n* = 5). Ultraviolet–visible (UV–vis) absorbance spectra of MX, Dox, and MD were recorded with a UV–vis spectrophotometer (S-3100, Scinco Co., Korea) to confirm the absorbance at 808 nm for NIR-responsive property. The optimized MD complex was further characterized by field-emission scanning electron microscopy (FE-SEM) (JSM-7800F, JEOL Ltd., Japan) and transmission electron microscopy (TEM; Talos L120C, Hillsboro, OR) to measure the size and morphology. The stability was assessed by monitoring the dispersion of MD in DI water with a charge-coupled device (CCD) camera (Canon EOS 100D; Canon, Japan) for 30 min, comparing the optimized sample with other ratios of MD formulations.

### Fabrication of MDGel

Each of the optimized MD complex (0.4 and 0.6 mg/ml) was mixed with the host component, GE-CD (200 mg/ml), in PBS for hydrogen bonding. The MD solution prepared in PBS was gradually added to the GE-CD solution with continuous stirring to ensure the formation of hydrogel intermediates by homogeneous interaction. Subsequently, the guest component, HA-AD (50 mg/ml), was physically mixed to the GE-CD/MD solution. The mixture was allowed to self-assemble by host–guest interactions between GE-CD and HA-AD, forming the MDGel system.

### Photothermal performance and rheological analysis

The PTT of MX, MD, and MDGel was assessed at the optimized concentrations of 0.4 and 0.6 mg/ml. Samples were prepared by adding 200 μl of each formulation into transparent cuvettes. The 808-nm laser was vertically irradiated onto the samples at 1.5 W/cm^2^ for 10 min, followed by a cooling period of 10 min. The temperature increase was recorded every 40 s with a thermal imaging camera (A3255C, FLIR), and the temperature profile was analyzed to confirm each sample to reach the temperature sufficient for cancer cell apoptosis. The injectable properties of MDGel were characterized by rheological analysis with an MCR 92 rheometer (Anton Paar, Torrance, CA). The storage modulus (*G*′) and loss modulus (*G*″) were measured to assess the viscoelastic properties critical for injection with a 25-mm parallel plate. Frequency sweeps were conducted at a strain of 1% over a range of 0.1 to 120 Hz. Based on these measurements, MDGel formulated at an MD concentration of 0.4 mg/ml was determined as the optimal hydrogel due to its superior injectability and its shear stress. The viscosity of selected MDGel was measured as a function of shear rate from 0.1 to 100/s.

### Degradation test

The degradation behavior of MDGel was evaluated over 7 d in 4 ml of PBS at 37 °C. At the predetermined time intervals, 200 μl of the solution was carefully sampled and the equivalent volume of PBS was replenished to maintain a consistent physiological environment. Optical density (OD) at 490 nm was measured for the quantitative tracking of Dox release during MDGel degradation with a microplate spectrophotometer (EMax End point ELISA microplate reader, Molecular Devices, Sunnyvale, CA) (*n* = 4).

### In vitro cytotoxicity and synergistic effect of PTT and chemotherapy

To evaluate the cytotoxicity and the synergistic therapeutic effect of PTT and chemotherapy, 200 μl of injectable supramolecular hydrogels was prepared for 4 groups: a control hydrogel (Gel), hydrogel encapsulating MX (MGel), hydrogel encapsulating Dox (DGel), and MDGel. Each hydrogel system was further divided into 2 subgroups based on the treatment conditions: with and without NIR irradiation. B16F10 murine melanoma cells were seeded in 96-well plates at a density of 1 × 10^5^ cells per well in DMEM supplemented with 10% FBS and 1% antibiotic–antimycotic solution. The cells were incubated in a humidified incubator at 37 °C with 5% CO_2_​ for 24 h. After incubation, for NIR-triggered subgroups, cells were irradiated with an 808-nm NIR laser at 1.5 W/cm^2^ for 10 min, while non-NIR subgroups were incubated under standard conditions and cultured for another 24 h. Afterward, the medium was replaced with a 10% CCK-8 solution and the cells were incubated for additional 4 h. OD at 450 nm was measured with a microplate reader (SoftMax, Molecular Devices) to assess the cell viability (*n* = 4). For live-dead staining, B16F10 cells were seeded at 1 × 10^5^ cells per well on confocal imaging plates. The medium was removed and treated with PBS, and then replaced with 200 μl of a staining solution containing calcein-AM (live cell marker) and ethidium homodimer-1 (dead cell marker). The cells were incubated for 30 min, and fluorescence images of live and dead cells were captured with a confocal laser scanning microscope (LSM 900 with Airyscan 2, Carl Zeiss).

### In vivo synergistic skin cancer therapy

A skin cancer model was established with 5-week-old male BALB/c mice. B16F10 melanoma cells (5 × 10^6^) were subcutaneously injected into both dorsal flanks of each mouse and allowed to develop for 7 d before treatment. The treatment groups and protocol mice were randomly assigned to 4 groups (*n* = 4) according to the injectable hydrogel formulations: Gel, MGel, DGel, and MDGel. For each mouse, 200 μl of the designated hydrogel formulation was injected to the tumor on both left and right flanks. The left flank was irradiated with an 808-nm NIR laser at 1.5 W/cm^2^ for 10 min to evaluate the photothermal effect, whereas the right flank served as the nonirradiated control. During the NIR treatment, temperature changes at the treatment site were monitored with a thermal imaging camera for the photothermal activation. Tumor size and body weight were tracked on days 0, 1, 2, 3, 5, and 7, and the tumor volume was calculated by the following formula:

$$\text{Volume}=\frac{\text{Length}\;\times \;{\left(\text{Width}\right)}^{2}}{2}$$ (1)

The treated areas were visually monitored and recorded with a CCD camera to assess the tumor progression and treatment efficacy. After 7 d of treatment, the mice were sacrificed, and tumors were excised and captured with a CCD camera to document visual differences among the treatment groups. Tumor tissues were fixed in 4% formaldehyde for the histological analysis. All animal experiments were conducted under protocols approved by the Institutional Animal Care and Use Committee (IACUC) of Pohang University of Science and Technology and followed the National Institutes of Health Guide for the Care and Use of Laboratory Animals.

### Histological analysis of tumor apoptosis

Following 7 d of in vivo treatment, the mice were sacrificed for histological analysis to evaluate tumor apoptosis and overall tissue morphology. Tumor tissues were carefully excised along with relevant organs and fixed in 4% formaldehyde for 24 h. The fixed tissues were subsequently dehydrated with a graded ethanol series (100%, 95%, 85%, 70%, and 50%) and embedded in paraffin. Paraffin blocks were sectioned into 5-μm slices with a microtome and mounted onto glass slides. The tumor sections were deparaffinized with xylene and rehydrated with descending ethanol concentrations for hematoxylin and eosin (H&E) staining. Sections were stained with hematoxylin for 5 min, rinsed, and counterstained with eosin for 2 min to visualize overall tissue morphology and tumor structure. For apoptosis, TUNEL assay was performed on paraffin-embedded tumor sections according to the manufacturer’s protocol. The apoptotic cells were visualized as red fluorescent signals under a 2-photon confocal microscope. The comparative analysis of apoptotic cells was conducted between treatment groups to evaluate the antitumor effect of MDGel following NIR irradiation.

### Statistical analysis

Statistical analysis was carried out via the *t* test using the software of SigmaPlot14.5 (Systat Software Inc., San Jose, CA). The values for *P < 0.05, **P < 0.01, and ***P < 0.005 were considered statistically significant. Data are expressed as means ± standard deviation (SD) from several separate experiments.

## Results

### Characteristics of supramolecular GE-CD/HA-AD hydrogel and MD complex

**Fig. 2.**
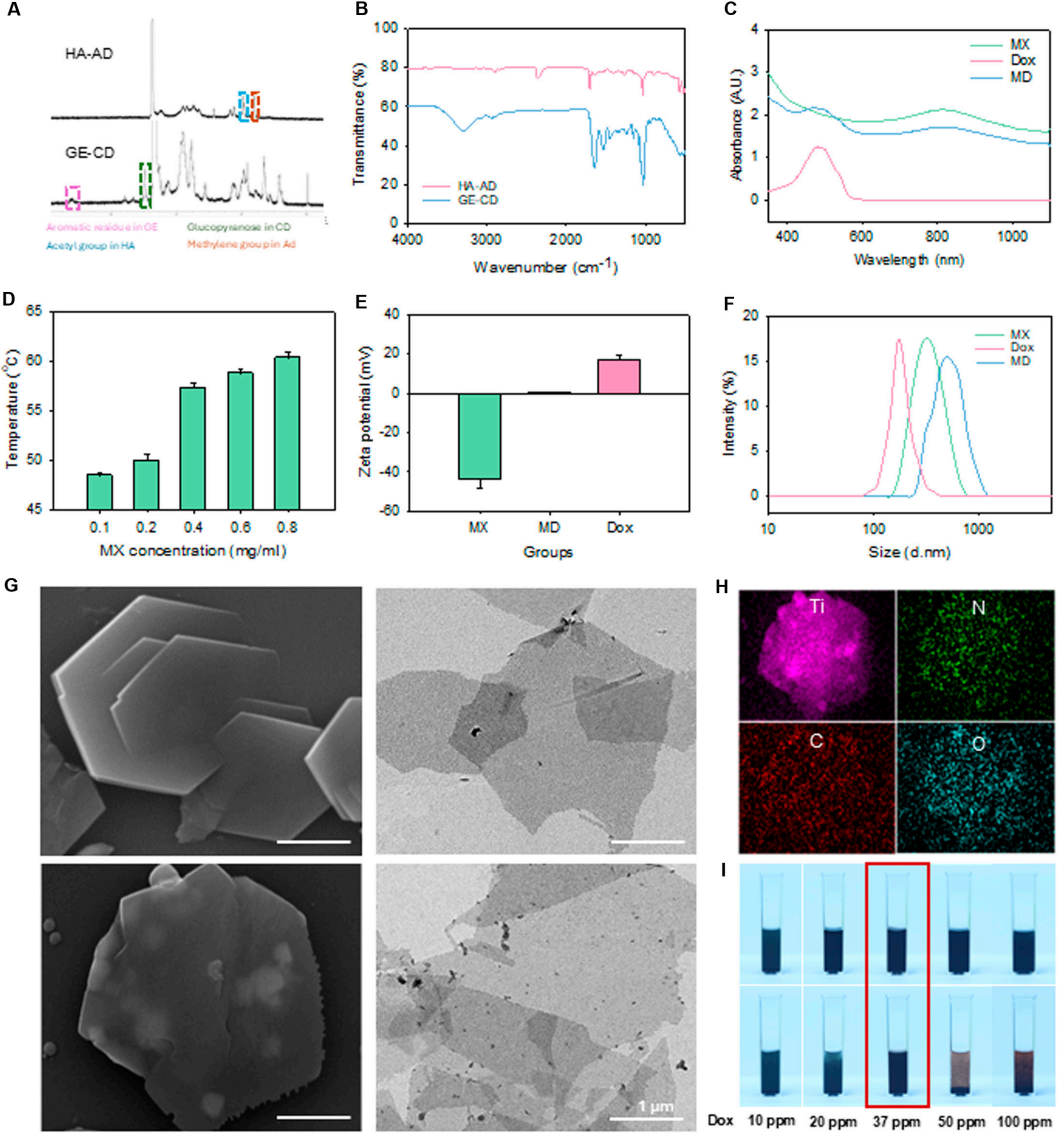
Characterization of GE-CD/HA-AD hydrogels and MD complexes. (A) ^1^H NMR spectra and (B) FT-IR spectra for the hydrogel components of HA-AD and GE-CD. (C) UV–vis spectra of MX, Dox, and MD complexes. (D) Photothermal effect of MX with the increasing concentration from 0.1 to 0.8 mg/ml. (E) Zeta potential and (F) size of MX (0.4 mg/ml), Dox (37 ppm), and MD complexes. (G) FE-SEM and TEM image of MX and MD complexes (0.4 mg/ml) and (H) SEM elemental mapping analysis of MD complexes. (I) Stability assessment of MD complexes prepared at the same MX concentration with varying Dox concentrations. The upper images represent MD complexes right after preparation, and the lower images show the same samples 30 min after incubation at room temperature. The red-marked image in the center represents the optimized MD complexes maintaining stable dispersion over time.

Injectable supramolecular hydrogels were successfully synthesized by the host–guest interaction between GE-CD and HA-AD. The self-assembled hydrogel exhibited rapid gelation upon mixing GE-CD and HA-AD solutions at a 1:2 ratio. The chemical structure and successful conjugation of GE-CD and HA-AD were confirmed by ^1^H NMR (Fig. 2A). The characteristic peak at δ = 5.0 to 5.3 parts per million (ppm) corresponded to β-cyclodextrin in GE-CD, and the peak at δ = 1.5 to 1.8 ppm corresponded to adamantane moieties in HA-AD. In addition, FT-IR spectroscopy confirmed the binding of CD and AD groups, with a decrease in transmittance at κ = 900 to 1,200/cm for GE-CD and the appearance of ester bond peaks at κ = 1,735 to 1,750/cm in HA-AD (Fig. 2B). The supramolecular hydrogel showed structural integrity with consistent host–guest assembly, providing a stable and injectable platform for subsequent integration with MD complexes. UV–vis absorbance spectra revealed the absorption peak of MX at 808 nm in the MD complexes (Fig. 2C). Additionally, the absorbance peak of Dox at 500 nm appeared in the MD complexes, reflecting the successful drug loading.

After that, the photothermal performance of MX was assessed by thermal imaging for 10 min with 808-nm NIR laser irradiation (Fig. 2D). The temperature of MX solutions was increased with increasing MX concentration. Especially, at the MX concentration of 0.4 mg/ml, the temperature was increased to 57 °C, indicating sufficient temperature increase for the effective tumor cell ablation [32,33]. Considering the thermal and mechanical properties, and the cytotoxicity, the MX concentrations of 0.4 and 0.6 mg/ml were selected for further experiments (Fig. S1A) [34,35]. The MD complex appeared stable via the electrostatic interaction. The zeta potential and size of MD (Fig. 2E and F and Fig. S1B) were optimized by varying the concentration of Dox, achieving a maximized Dox loading on the MX. After that, we confirmed the layered structure of MD complexes by TEM and SEM (Fig. 2G and H). The EDS analysis further validated the successful drug loading since nitrogen, an element of Dox, was detected in the MD complex. As shown in Fig. 2I, the MX precipitated over time at low Dox concentrations, whereas MX was aggregated at high Dox concentrations. However, in the optimal concentration of Dox, the MD complexes were well dispersed in water for over 30 min without aggregation or sedimentation. These results corroborated the stability of MD and its suitability as a pre-crosslinking agent for injectable hydrogels.

### Photothermal and mechanical properties of MDGel

**Fig. 3.**
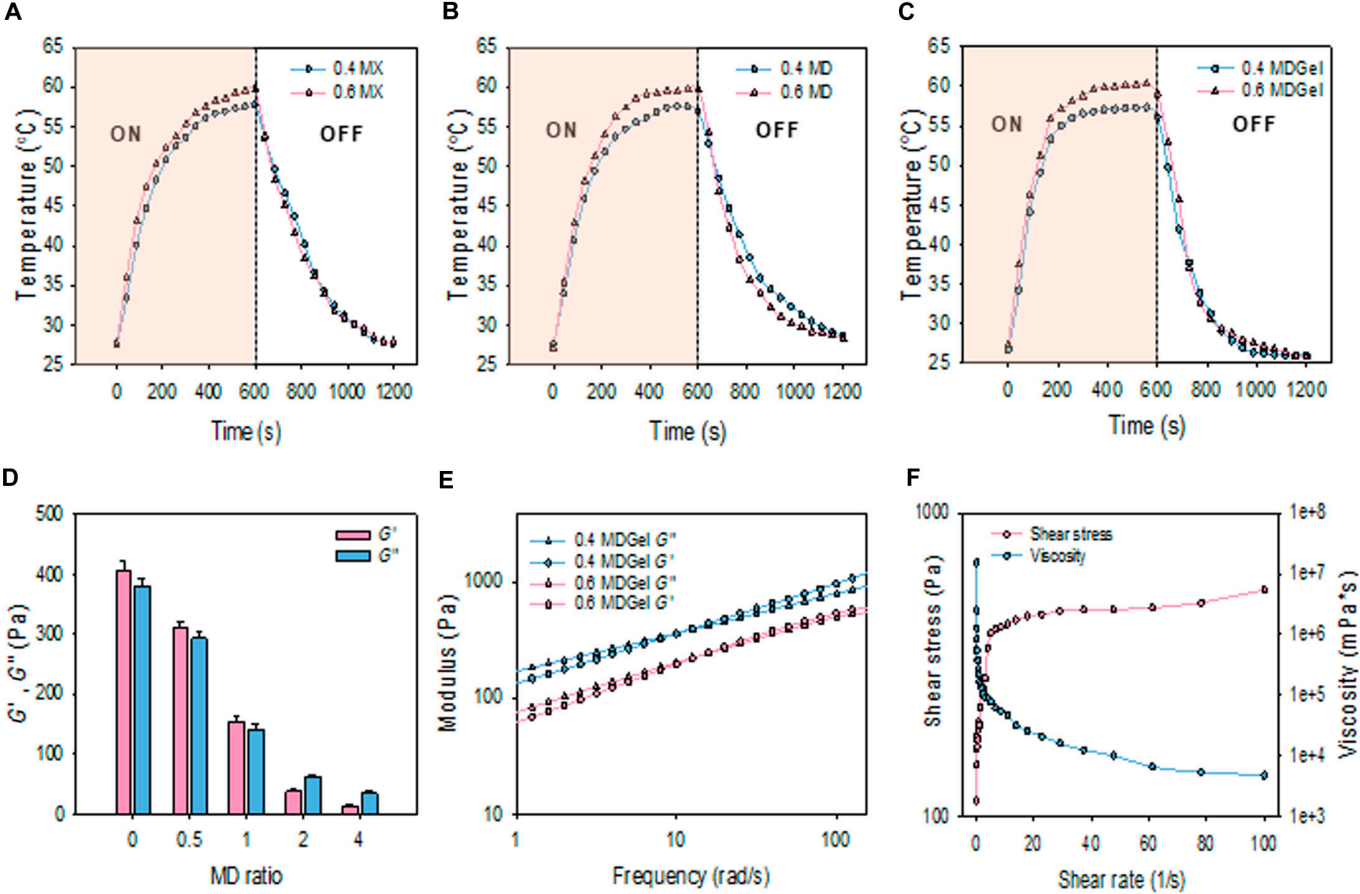
Preparation of MDGel. The photothermal performance at the selected concentrations (0.4 and 0.6 mg/ml) of (A) MX, (B) MD, and (C) MDGel. (D) Storage moduli (*G*′) and loss moduli values of MDGel prepared with GE-CD and HA-AD (1:2 ratio) as a function of the MD ratio. (E) Frequency sweep analysis of MDGel. (F) Continuous flow analysis of the optimized MDGel (0.4 mg/ml) (0.1 to 100/s).

The photothermal effect of MX, MD, and MDGel was assessed at 0.4 and 0.6 mg/ml (Fig. 3A to C). The temperature profiles showed the typical photothermal behavior in all groups. At the MX concentration of 0.4 and 0.6 mg/ml, the temperature was increased to 56 and 58 °C. In the case of MD and MDGel, the temperature was increased to 54 °C upon NIR light irradiation, indicating that the drug loading and hydrogel integration did not significantly compromise the photothermal property of MX. As shown in the inset of Fig. 3C, MDGel was visibly degraded under NIR irradiation or heating, exhibiting the thermal responsiveness of supramolecular hydrogel. This property enhanced the potential for on-demand drug release triggered by localized heating.

The mechanical property of MDGel was analyzed to evaluate its injectability of the MDGel system. In the modulus measurements, the crossover point for the storage modulus and the loss modulus was measured to find the optimal gelation ratio (Fig. 3D). From the results, the optimal gelation ratio of GE-CD:HA-AD:MD was determined to be 1:2:1 with the balanced elasticity and structural integrity for hydrogel stability. The formulation at 0.4 mg/ml showed a relatively higher *G*′ value (147.2 Pa) than that at 0.6 mg/ml (60.8 Pa) at low frequencies, denoting superior structural stability under minimal stress (Fig. 3E). Considering the storage moduli of soft biological tissues, MDGel with MX at 0.4 mg/ml was chosen for further experiments. Continuous flow experiments showed the shear-thinning behavior of MDGel and Gel without MD complexes, demonstrating its injectability through a needle (Fig. 3F and Fig. S2). The optimized MD complexes were successfully encapsulated into the GE-CD/HA-AD hydrogel matrix with the homogeneous dispersion of MD complexes. This stepwise assembly process resulted in the robust injectable hydrogel preparation with consistent mechanical properties.

### In vitro release, cytotoxicity, and antitumor effect of MDGel system

**Fig. 4.**
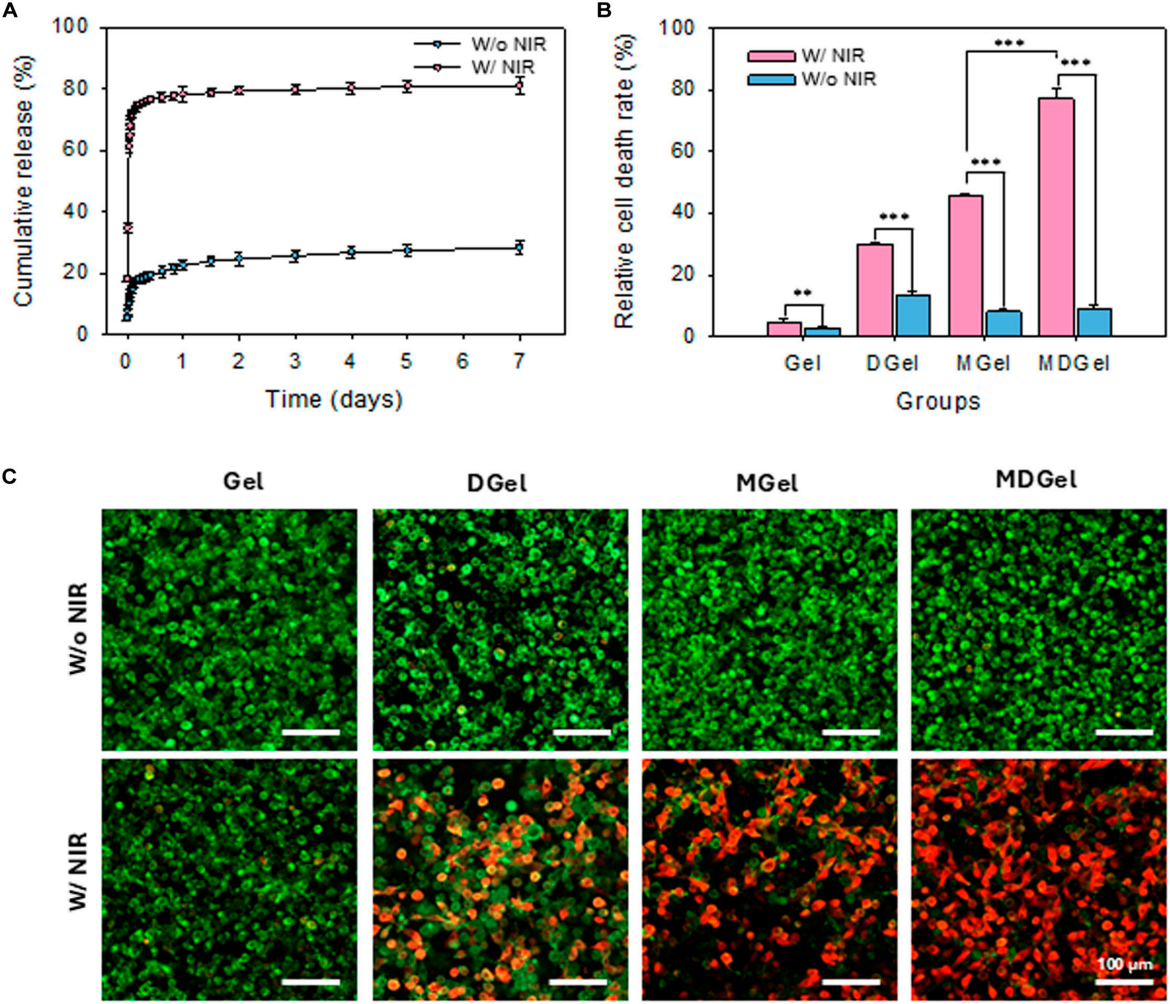
In vitro release and antitumor effect of MDGel. (A) Dox release profile from MDGel for 7 d under physiological conditions (PBS, 37 °C) with and without NIR irradiation (808 nm, 1.5 W/cm^2^, 10 min). (B) Cell viability in B16F10 melanoma cells after treatment of various hydrogel formulations (Gel, MGel, DGel, and MDGel) with and without NIR irradiation via the CCK-8 assay. (C) Live/dead fluorescence staining of B16F10 cells following the hydrogel treatment (**P < 0.01 and ***P < 0.005).

Figure 4A shows in vitro release of Dox from MDGel in PBS at 37 °C for 7 d. In the NIR-treated group, Dox was significantly released from the MDGel with a rapid release for the first 24 h and the saturation at 81.2 ± 3.0%. In contrast, only 28.6 ± 2.3% of Dox was released in the non-NIR-treated group. These results highlighted the temperature responsive disintegration of MDGel, facilitating controlled drug release upon NIR stimulation. After that, cell viability was assessed by the CCK-8 assay (Fig. 4B). The MDGel group with NIR irradiation showed the highest cytotoxicity with 77.2 ± 2.9% cancer cell death, indicating the synergistic effect of photothermal effect of MX and the triggered release of Dox. The synergistic effect of combining PTT and chemotherapy in the MDGel resulted in significantly enhanced cytotoxicity compared to single-modality treatments of MGel (45.7 ± 0.6%) and DGel (30.1 ± 0.3%), reflecting the advantages of the dual-functional hydrogel system with NIR irradiation. The live-dead staining of B16F10 cells also confirmed the best therapeutic effect of MDGel with NIR irradiation on the cancer cells (Fig. 4C and Fig. S3). The MDGel with NIR showed the significant cell death with almost complete red fluorescence coverage, confirming the feasibility of the MDGel system for NIR light controllable therapeutic platform.

### In vivo antitumor effect of MDGel

**Fig. 5.**
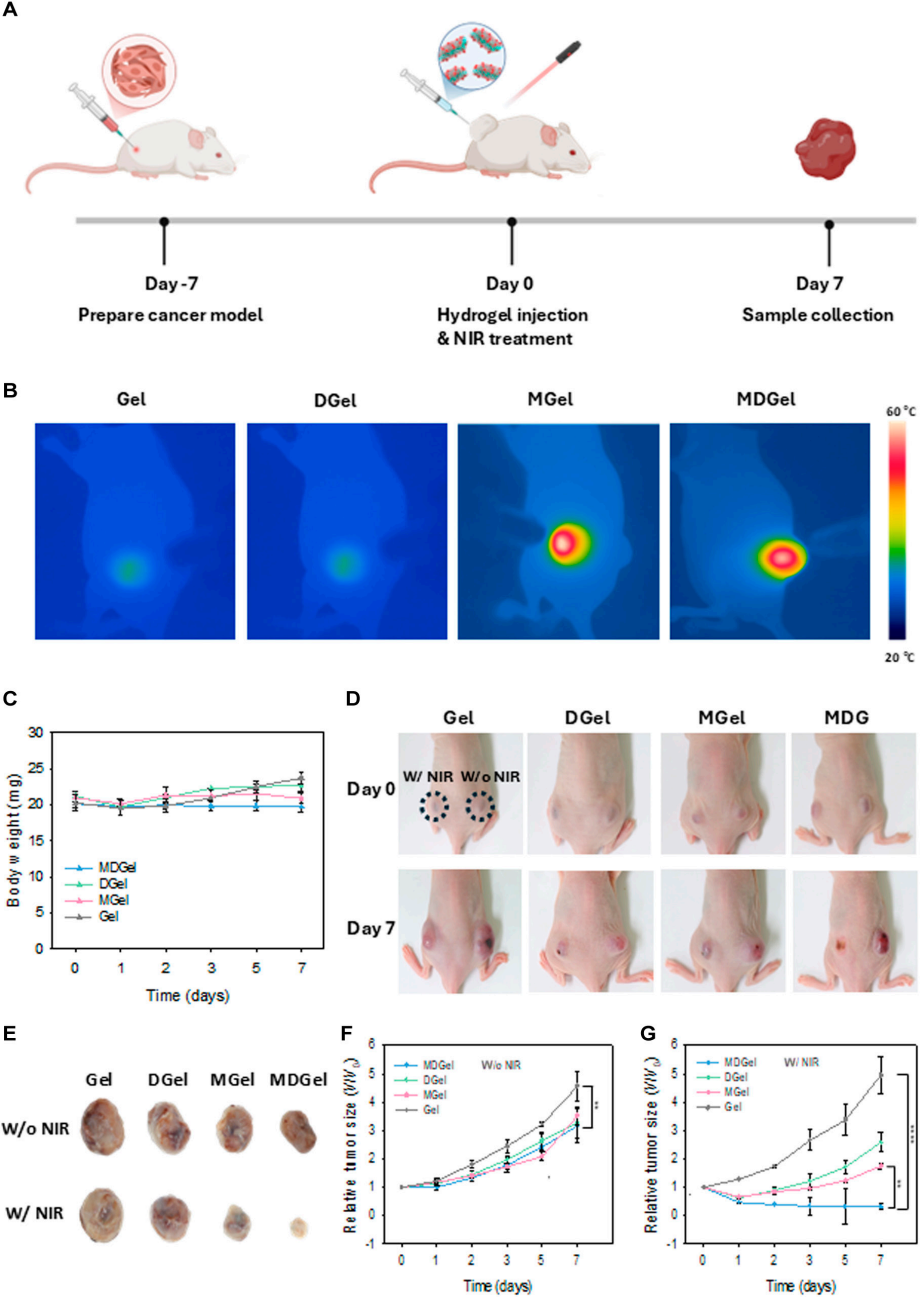
In vivo synergistic skin cancer therapy. (A) Schematic illustration for the cancer therapy schedule with the MDGel system. B16F10 melanoma cells were subcutaneously injected into both dorsal flanks of BALB/c mice, and the tumor samples were collected on day 7 post-treatment (created with BioRender.com). (B) Thermal image of tumor sites after NIR irradiation (808 nm, 1.5 W/cm^2^) for 10 min. (C) Body weight change during the treatment. (D) Representative photographs of tumor-bearing mice dissected from each group (Gel, MGel, DGel, and MDGel) on days 0 and 7. Tumors on the left flank were treated with NIR irradiation (808 nm, 1.5 W/cm^2^, 10 min) following the hydrogel injection, whereas those in the right flank were used as a nonirradiated control. (E) Final excised tumor photographs on day 7 and the relative volume of tumors after therapy for 7 d (F) without and (G) with NIR irradiation (**P < 0.01 and ****P < 0.001).

The therapeutic efficacy of the novel MDGel system was evaluated in skin cancer-bearing mice model. A dorsal flank tumor model was captured throughout the experimental process with camera (Fig. S4A) and established in mice to rigorously evaluate the antitumor efficacy of MDGel under NIR irradiation (Fig. 5A). Thermal imaging revealed the photothermal effect of MDGel at the tumor site (Fig. 5B). Groups containing MX exhibited significant temperature increases above 55 °C in the tumor site, demonstrating the effective PTT upon NIR light irradiation. In addition, the MDGel group maintained stable body weight throughout the treatment period, meaning the biocompatibility and minimal off-target effects of the MDGel system (Fig. 5C). Moreover, NIR-treated mice without injectable hydrogel exhibited negligible tissue damage, indicating moderate intensity of NIR light for PTT (Fig. S5A). As shown in Fig. 5D to G and S4B, tumors were monitored for 7 d for all treatment groups. In the non-NIR-treated groups, the tumor inhibition effect was not clearly observed in MGel, DGel, and MDGel groups due to the minimal Dox release and negligible photothermal effect. In contrast, in the NIR-treated groups, the MGel and DGel groups exhibited limited therapeutic efficacy based solely on the PTT and chemotherapeutic action, respectively. Meanwhile, the MDGel group exhibited the most significant tumor size reduction with a near-complete regression of the tumor (68.9 ± 6.9% of tumor reduction). The results demonstrated the remarkable efficacy of the MDGel in targeted, externally controlled cancer therapy.

### Histological analysis

**Fig. 6.**
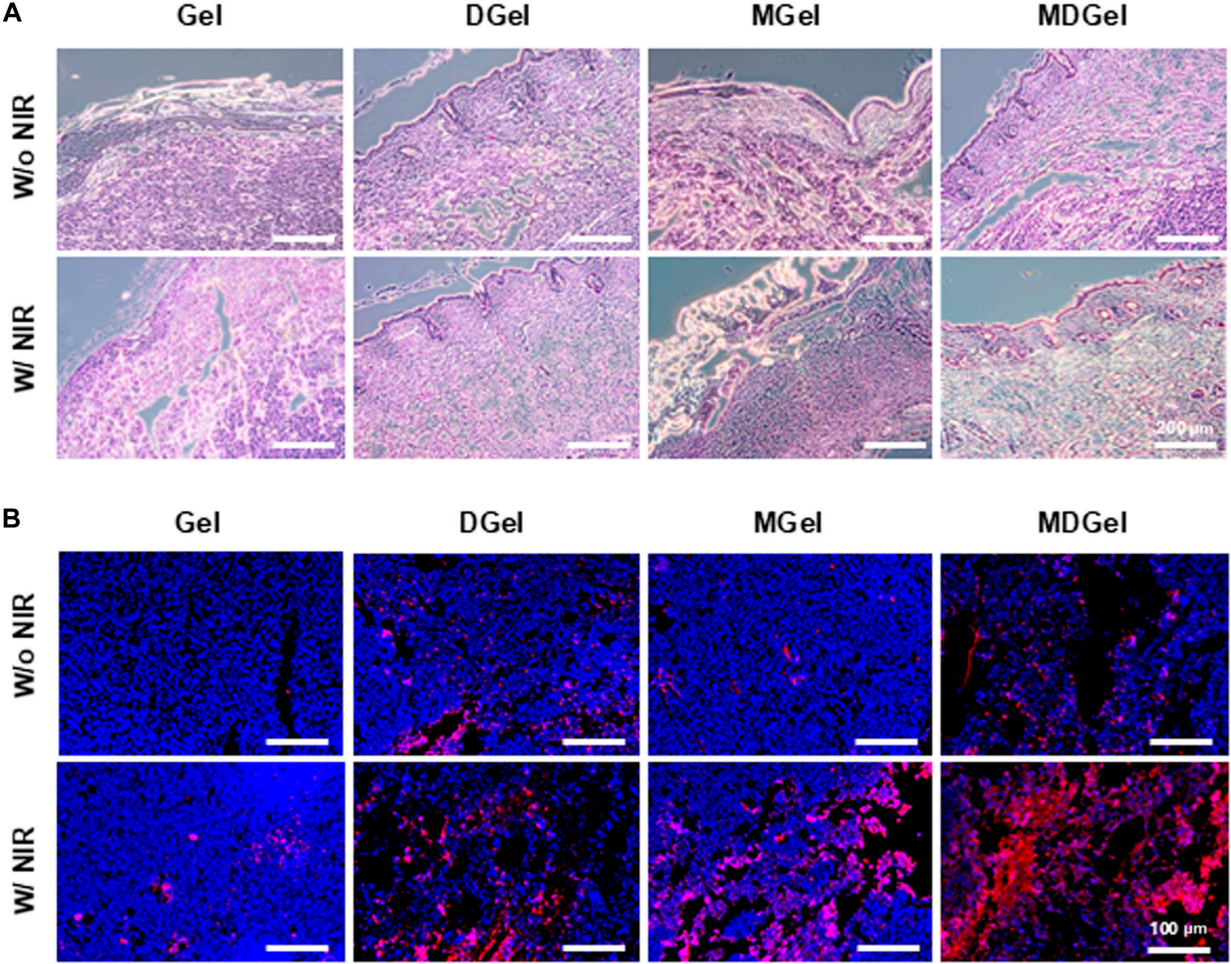
Histological analysis of paraffin-embedded tumor tissues. (A) H&E staining and (B) TUNEL assay for each hydrogel formulation and treatment condition.

Histological evaluation with H&E staining revealed significant differences in tumor morphology between NIR-treated and nontreated groups (Fig. 6A). In the NIR-treated groups, enhanced drug release and photothermal effect led to pronounced tumor ablation in the MDGel group, demonstrating the most substantial reduction in tumor cell density and widespread necrosis compared to other treatment groups. Tumor tissues in the MDGel with NIR group exhibited visible structural degradation, indicative of synergistic PTT and chemotherapeutic activity, whereas single-modality treatment groups showed partial tumor cell loss with less extensive necrotic regions. Furthermore, while localized tissue damage was observed in NIR-exposed areas, significant inflammatory responses were not detected in the nontreated region, suggesting minimal off-target toxicity. TUNEL analysis corroborated the H&E findings by carrying out extensive apoptotic cell populations in the MDGel with NIR group. Quantitative fluorescence imaging confirmed a significantly higher number of apoptotic cells in the tumor microenvironment where the apoptotic cells were predominantly concentrated within the core and periphery of tumor masses (Fig. 6B and Fig. S5B). The distribution and density of apoptotic cells were markedly higher in the MDGel with NIR group compared to the MGel and DGel groups, reinforcing the synergistic therapeutic efficacy of the dual PTT and chemotherapy system. The results validated the potent antitumor effect of the MDGel system, demonstrating its ability to induce substantial tumor regression with minimal adverse effects for externally controlled cancer therapy.

## Discussion

The MDGel system demonstrated a promising NIR light controllable therapeutic platform highlighting the key advancements in hydrogel-based cancer therapies, including the development of a dual synergistic therapeutic platform, effective stimuli-responsive behavior, and robust biocompatibility. In this system, effective integration of MD complexes into a supramolecular hydrogel matrix ensured the stability, precise targeting, and sustained retention at the tumor site. The dynamic host–guest interactions between GE-CD and HA-AD formed a structurally stable platform capable of withstanding physiological conditions while preventing the premature release of therapeutic agents. This stability was further confirmed by in vivo experiments in which the MDGel exhibited therapeutic effect only under NIR light irradiation. Extensive necrosis and reduced tumor cell density in the MDGel with NIR indicated the superior tumor ablation compared to single-modality groups, maximizing tumor cell apoptosis, and minimizing residual tumor tissue. In vivo monitoring illustrated that the MDGel with NIR achieved near-complete tumor regression by day 7, whereas single-modality groups exhibited only partial tumor suppression. The absence of significant inflammatory responses in the treatment groups underscored the biocompatibility and safety of MDGel. TUNEL immunohistochemistry further corroborated the histological findings by quantifying apoptotic cell populations within the tumor microenvironment, demonstrating superiority of dual-modality therapy in achieving complete tumor eradication. In addition, the GE within the hydrogel matrix facilitated post-treatment tissue recovery and minimized scarring, addressing a critical limitation associated with thermal destruction techniques.

The MDGel system holds significant promise as a versatile biomedical platform for the localized cancer therapy. Its ability to combine PTT and chemotherapy with prolonged retention, stability, and NIR-responsive controlled release would offer a unique advantage for addressing critical challenges such as cancer recurrence. After initial tumor treatment, MDGel could remain at the site of potential relapses, delivering therapeutic agents on-demand to target residual or emerging cancer cells and reduce recurrence rates. Further functionalization with immune modulators, anti-angiogenic agents, or small interfering RNA (siRNA) would enhance its preventive capabilities by inhibiting tumor regrowth or stimulating immune responses. Additionally, MDGel’s dynamic host–guest interactions and tunable properties would make it adaptable for multimodal therapies, integrating PDT with imaging agents, enabling real-time monitoring and optimized therapy. Beyond oncology, the platform’s versatility would support for the chronic inflammatory disease treatment, such as localized drug delivery for rheumatoid arthritis or inflammatory bowel disease, where controlled and sustained release of anti-inflammatory agents is critical. Furthermore, MDGel would be utilized in regenerative medicine by incorporating growth factors or stem cells for tissue engineering applications, such as cartilage repair or wound healing. The system’s adaptability also extends to antibacterial therapies by loading antibiotics or antimicrobial peptides for localized infection management, particularly in wound care or surgical site infections. This multifunctional MDGel would be harnessed as a transformative tool for advancing precision medicine across diverse biomedical applications.

In conclusion, we successfully developed MDGel as a synergistic platform for cancer therapy. By the dynamic host–guest interaction between GE-CD and HA-AD encapsulating MD complexes, this supramolecular hydrogel achieved enhanced stability, prolonged retention, and controlled drug release. In this system, the hydrogel consistently elevated temperatures to approximately 56 °C under NIR irradiation, enabling effective PTT with controlled drug release (81.2 ± 3.0% for 7 d). In vitro studies demonstrated 77.2 ± 2.9% cancer cell death in the MDGel with NIR group, significantly outperforming single-modality treatments (MGel: 45.7 ± 0.6%, DGel: 30.1 ± 0.3%). Furthermore, the group of MDGel with NIR showed 68.9 ± 6.9% tumor volume reduction over 7 d in skin cancer-bearing mice model with almost complete regression. Histological analysis also revealed the extensive necrosis and reduced tumor cell density without notable inflammatory responses, highlighting the minimal off-target effects. Taken together, this versatile and minimally invasive platform holds great promise for further clinical applications, offering a new paradigm for externally controlled, site-specific cancer therapy.

## Data Availability

All data presented in this manuscript, including Supplementary Materials, are available from the corresponding authors upon reasonable request.
