# Supplementary material for: MXene/Doxorubicin Complex-Loaded Supramolecular Hydrogels for Near Infrared-Triggered Synergistic Cancer Therapy
Source: Biomater Res. 2025 Apr 9;29:0163. doi: 10.34133/bmr.0163 (PMC11979340; doi:10.34133/bmr.0163)
Supplement: Supplementary 1 — Figs. S1 to S5 [file bmr.0163.f1.zip › BiomaterRes-Supplementary-Hydrogel.pdf]

SCIENCE PARTNER JOURNALS

Manuscript Template

Supplementary Materials

MXene/Doxorubicin Complex Loaded Supramolecular Hydrogels for NIR-Triggered Synergistic Cancer Therapy

Seung Min Yang, Hanseo Bae, Seong-Jong Kim, Mungu Kim, Sang Hoon Hong, Hyunsik Choi* and Sei Kwang Hahn*

Department of Materials Science and Engineering, Pohang University of Science and Technology (POSTECH), 77 Cheongam-ro, Nam-gu, Pohang, Gyeongbuk 37673, Republic of Korea.

***CORRESPONDING AUTHOR FOOTNOTE**

Tel.: +82 54 279 1759; Fax: +82 54 279 2399; E-mail: chlgustlr67@postech.ac.kr (H. Choi)

Tel.: +82 54 279 2159; Fax: +82 54 279 2399; E-mail: skhanb@postech.ac.kr (S.K. Hahn)

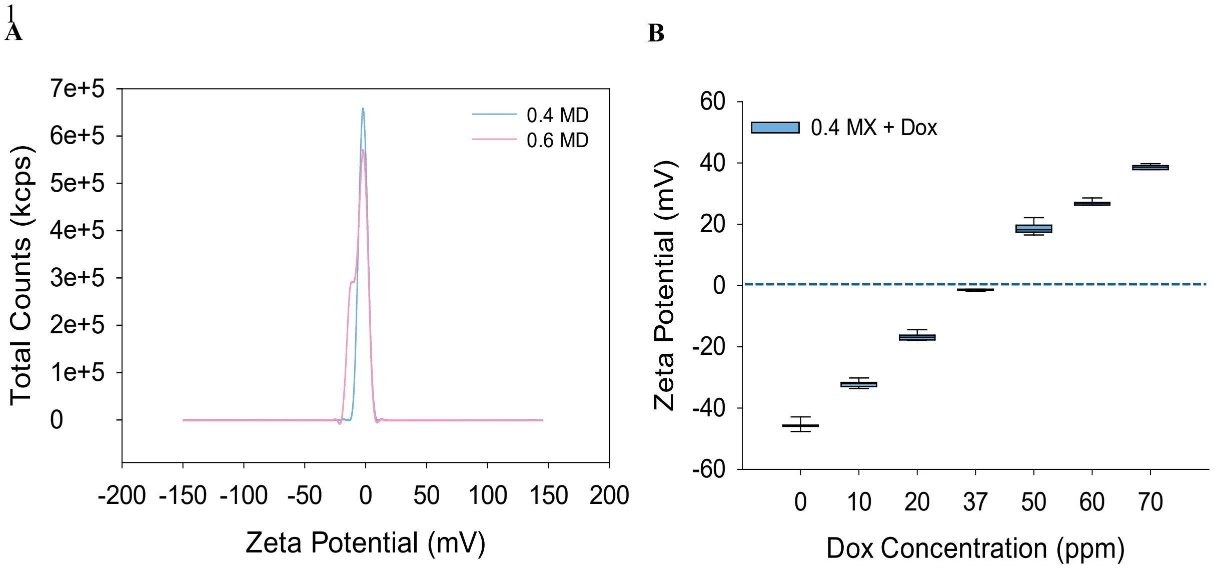

Fig. S1. (A) Zeta potential of MD complexes at the optimized MX concentration of 0.4 and 0.6 mg/mL. (B) Zeta potential of MD complexes at the selected MX concentration of 0.4 mg/mL with increasing concentration of Dox.

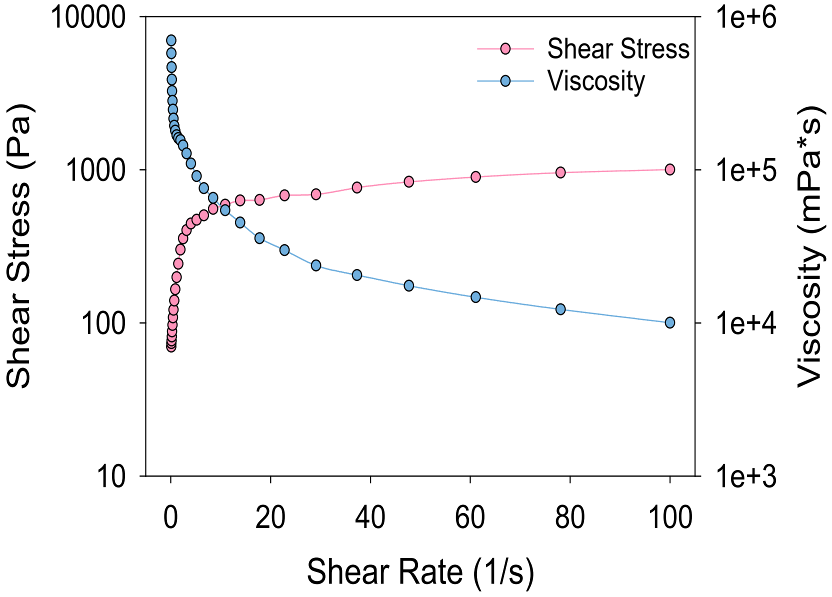

Fig. S2. Continuous flow analysis for the optimized supramolecular hydrogel of GE-CD and HA-AD (1:2 ratio) without MD complexes.

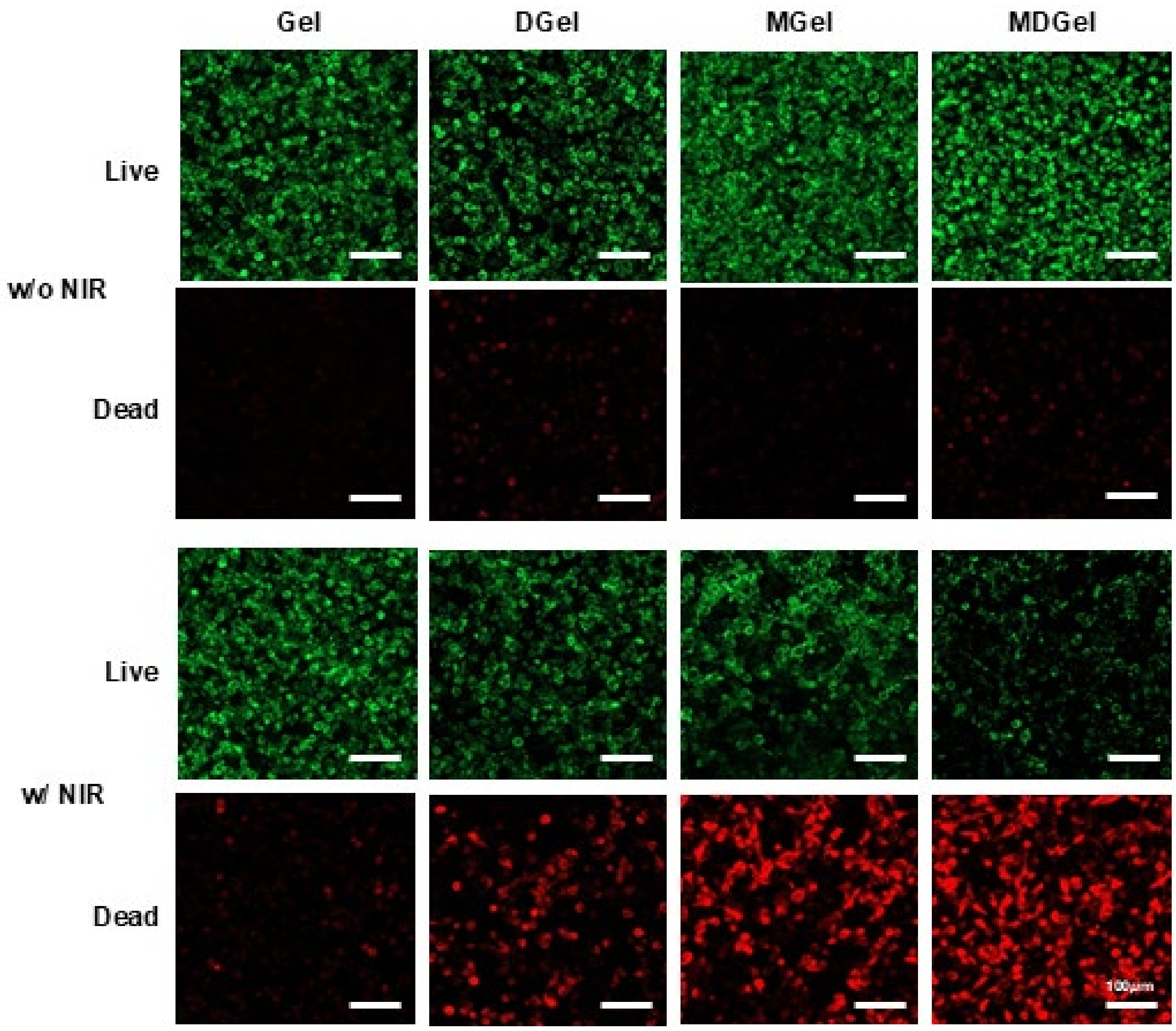

Fig. S3. Fluorescence images of live cells stained with calcein-AM (green) and dead cells stained with ethidium homodimer-1 (red) for each treatment group. Images show individual channels of live and dead cells prior to merging.

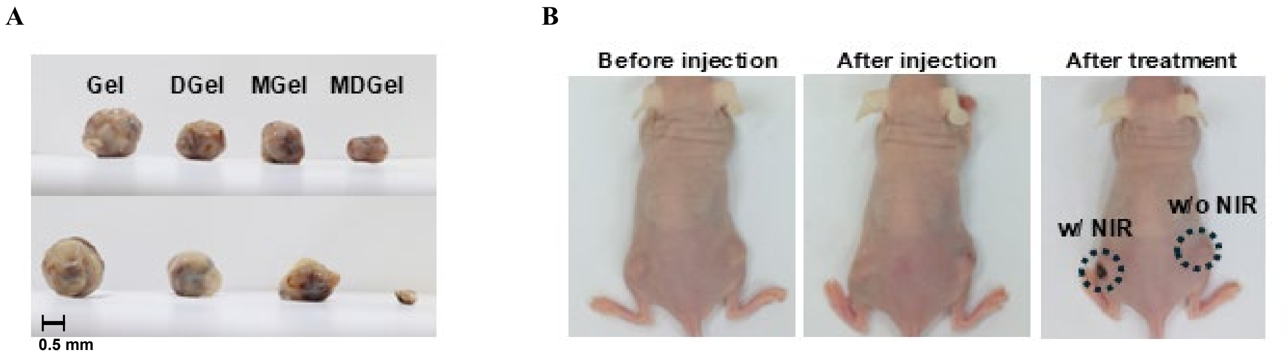

Fig. S4. (A) Photo-images of excised tumors from each treatment group 7 days post-treatment. Tumors are shown in both lying and upright orientations to clearly show the differences in size and morphology. (B) Photo-images of model mice pre-injection, post-injection and after treatment (left flank) with MDGel and NIR light irradiation.

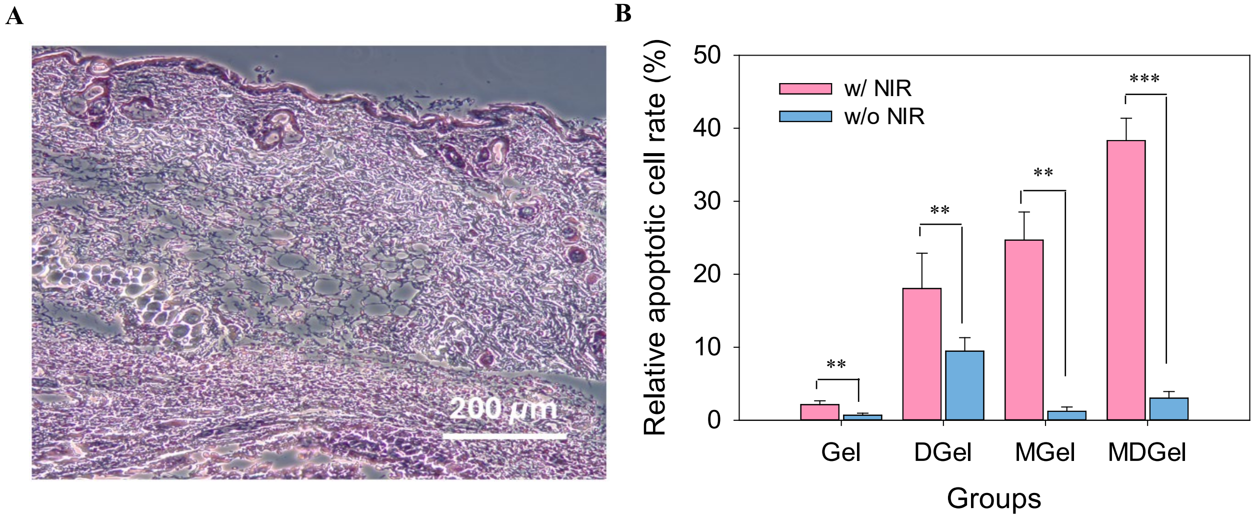

Fig. S5. (A) Histological image with H&E staining for NIR treated mouse (808 nm, 1.5 W/cm², 10 min) without the injectable hydrogel. (B) Quantification for the relative apoptotic cell rate from TUNEL assay for each different formulation group (**P < 0.01 and ***P < 0.005).
