# Supplementary figures and images for: MXene/Doxorubicin Complex-Loaded Supramolecular Hydrogels for Near Infrared-Triggered Synergistic Cancer Therapy
Source: Biomater Res. 2025 Apr 9;29:0163. doi: 10.34133/bmr.0163 (PMC11979340; doi:10.34133/bmr.0163)

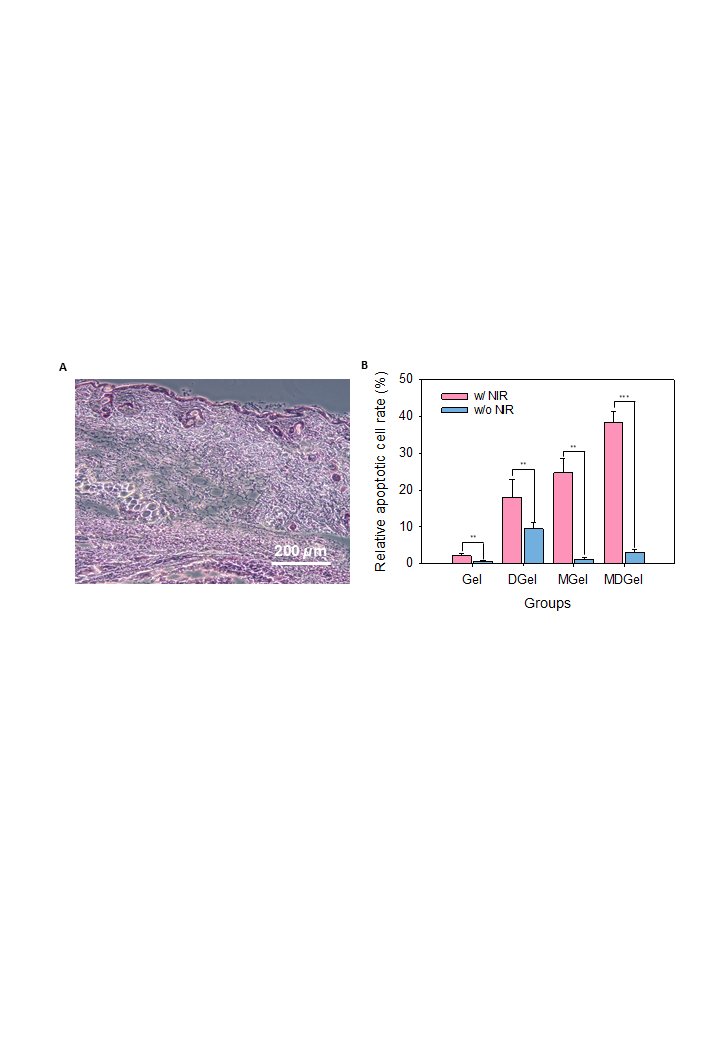

Supplement: Supplementary 1 — Figs. S1 to S5 [file bmr.0163.f1.zip › Fig. S5.PNG]
